# Robust dynamics in minimal hybrid models of genetic networks

**DOI:** 10.1098/rsta.2010.0139

**Published:** 2010-11-13

**Authors:** Theodore J. Perkins, Roy Wilds, Leon Glass

**Affiliations:** 1 Ottawa Hospital Research Institute, 501 Smyth Road, Ottawa, Ontario, Canada K1H8L6; 2 Department of Mathematics and Statistics, McGill University, 805 Sherbrooke Street West, Montreal, Quebec, Canada H3A2K6; 3 Department of Physiology, McGill University, 3655 Promenade Sir William Osler, Montreal, Quebec, Canada H3G1Y6

**Keywords:** genetic networks, symbolic dynamics, robustness, nonlinear dynamics, yeast cell cycle

## Abstract

Many gene-regulatory networks necessarily display robust dynamics that are insensitive to noise and stable under evolution. We propose that a class of hybrid systems can be used to relate the structure of these networks to their dynamics and provide insight into the origin of robustness. In these systems, the genes are represented by logical functions, and the controlling transcription factor protein molecules are real variables, which are produced and destroyed. As the transcription factor concentrations cross thresholds, they control the production of other transcription factors. We discuss mathematical analysis of these systems and show how the concepts of robustness and minimality can be used to generate putative logical organizations based on observed symbolic sequences. We apply the methods to control of the cell cycle in yeast.

## 1. Introduction

Biological systems display remarkably robust dynamics. This robustness is evident at levels of organization from the cell to the organism. Indeed, despite the evolution of genetic control circuits, key structures and organs function in similar fashions in different organisms. Understanding the principles underlying such phenomena remains an important challenge.

We believe that hybrid systems may help provide a conceptual and computational basis for these observations. Hybrid systems combine aspects of discrete, logical systems and continuous systems. In molecular biology, it is common to speak of genes being switched on or off, and in some organisms there is substantial experimental evidence demonstrating precise sequences of gene activation in time and space (Mjolsness *et al.* 1991; Jaeger *et al.* 2004). Thus, a natural approach to the study of genetic control conceives of genes as being switched on or off depending on the concentrations of key control variables. Typically, these are specialized protein molecules called transcription factors. A significant literature adopts this approach. For a review, see De Jong (2002).

The idealization of genes as logical elements does not provide an immediate basis for robust dynamics. In typical discrete Boolean switching networks, many elements simultaneously change their state (Kauffman 1969). Such behaviour is not generally robustly preserved in systems with stochastic updating, or in differential equation models (Bagley & Glass 1996). However, by embedding genetic logic in continuous differential equations, it is possible to provide results that demonstrate criteria for the logical structure of networks that will guarantee robust dynamics.

The basic idea is to subdivide continuous phase space into a finite number of volumes. The flows between these volumes can be represented by a directed graph. In our formulation, this directed graph, which we call the *state transition diagram*, provides the essential device to link the underlying logical structure of networks and the dynamics. To generate robustness, we make an ansatz that the dynamics in the state transition diagram are focused, so that from any given vertex there is a single next state. Such an assumption forms the basis for earlier analyses of stable oscillations and computation in model genetic networks (Glass & Pasternack 1978*b*; Ben-Hur & Siegelmann 2004).

In the setting of the hybrid dynamical systems we use to model genetic networks, the natural structure of the state transition diagram is a directed *N*-dimensional hypercube (*N*-cube). In previous work, we have described many of the essential features of these equations. We have demonstrated instances in which the *N*-cube representation can be used to predict qualitative features of the dynamics, including fixed points and oscillations. We and others have described methods for solving the inverse problem—that is, for determining the underlying logical structure based on the observed dynamics. Further, defining the *minimal network* to be a network in which each variable has the fewest number of inputs, we determined minimal networks generating robust stable limit cycle oscillations and applied these results to networks with up to five variables. Our point in the following is to briefly summarize the main results, to specifically discuss features that guarantee both robustness and minimality and to show how these methods can be applied to determine a hybrid system consistent with observed dynamics for control of the cell cycle in yeast.

## 2. Model equations

We model the dynamics of a system of *N* continuous variables, 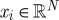. In applications, the *x*_*i*_ are typically taken to be the concentrations of the proteins encoded by genes (De Jong 2002). In principle, the *x*_*i*_ might represent many other things, such as relative concentrations, the fraction of molecules of some species that are bound by a modifying molecule, or the fraction of time that a single molecule is in one conformation or another. For convenience of exposition, we will refer to the *N* things being modelled as the concentrations of different chemical species, even though this language does not cover all the possibilities above.

To each variable *x*_*i*_ we also associate a discrete, or qualitative, state, *X*_*i*_, depending on whether *x*_*i*_ is above or below a threshold value *θ*_*i*_: *X*_*i*_ = 1 if *x*_*i*_ ≥ *θ*_*i*_, and *X*_*i*_ = 0 if *x*_*i*_ < *θ*_*i*_. The dynamics of the system are given by
2.1
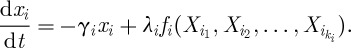

Here, *γ*_*i*_ > 0 represents the rate at which the concentration of species *i* decays, which may be due to actual degradation of molecules or to dilution as a result of cell growth. *λ*_*i*_ > 0 represents the maximum rate at which species *i* can be generated, and *f*_*i*_: {0,1}^*k*_*i*_^↦{0,1} is a *k*_*i*_-input Boolean function called the regulatory function. Further, we assume that *λ*_*i*_/*γ*_*i*_ > *θ*_*i*_ > 0 so that the maximal production of each species will be adequate to be superthreshold.

The dynamics of species *i* depend only on its own concentration for the decay term, and on the qualitative states of *k*_*i*_ ≤ *N* species, which are called the regulators of species *i*. This set of regulators is denoted by *R*_*i*_ = {*i*_1_,*i*_2_,…,*i*_*k*_*i*__}. The sets of regulators of every species, (*R*_1_,*R*_2_,…,*R*_*N*_), and the Boolean functions controlling production in equation (2.1), (*f*_1_,*f*_2_,…,*f*_*N*_), are termed the logical structure of the system. We will further assume that *i*∉*R*_*i*_. This no self-input condition has important technical consequences that we make clear in the next section. For practical applications in which genes do appear to regulate their own activity, it is possible to introduce a new variable which is only activated by the gene in question, and to have that new variable in turn activate the gene. We assume that all regulators are *effective*, meaning that, for every *i* and for every *j*∈{1,2,…,*k*_*i*_}, there is at least one setting for all the other regulators, *X*_*i*_1__,…,*X*_*i*_*j*−1__,*X*_*i*_*j*+1__,…,*X*_*i*_*k*_*i*___, such that *f*_*i*_(*X*_*i*_1__,…,*X*_*i*_*j*−1__,0,*X*_*i*_*j*+1__,…,*X*_*i*_*k*_*i*___) ≠ *f*_*i*_(*X*_*i*_1__,…,*X*_*i*_*j*−1__,1,*X*_*i*_*j*+1__,…,*X*_*i*_*k*_*i*___).

In more general formulations of the model, each species may be associated with *M* ≥ 2 possible qualitative states defined by a set of *M* − 1 thresholds, or the decay and production rates *γ*_*i*_ and *λ*_*i*_ may also depend on the qualitative states of other species (De Jong 2002). The formulation above, however, is the one that has been most commonly used in mathematical analyses of dynamics and reverse engineering, and is sufficient for the purposes of this paper.

Equation (2.1) describes a piecewise linear system of ordinary differential equations. In particular, the *θ*_*i*_ collectively define 2^*N*^ orthants of phase space—subsets of 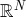
—inside each of which the qualitative state of every species is the same. Inside each orthant, the dynamics of each species *i* follow either d*x*_*i*_/d*t* = − *γ*_*i*_*x*_*i*_ or d*x*_*i*_/d*t* = − *γ*_*i*_*x*_*i*_+*λ*_*i*_, depending on the value of *f*_*i*_ for that orthant. Thus, in every orthant the dynamics are focused in the sense that the trajectory would asymptotically approach a focal point, with coordinates (*f*_1_*λ*_1_/*γ*_1_,…,*f*_*N*_*λ*_*N*_/*γ*_*N*_). However, if a threshold of one of the variables is transgressed, then the values of one or more of the *f*_*i*_ may change suddenly. This causes the focal point to change, leading to corners on the trajectory. Earlier papers have described the diverse repertoire of different possible behaviours, including convergence to a fixed point, periodic orbits, quasi-periodic orbits and chaos (Glass & Pasternack 1978*b*; Edwards & Glass 2005).

The discontinuity of the right-hand side as a function of **x** = (*x*_1_,*x*_2_,…,*x*_*N*_) means that solutions are not in general guaranteed to exist or to be well-defined in the usual sense, nor are they necessarily unique. For a rigorous treatment, one recourse is to redefine the dynamics equations as differential inclusions and to consider solutions in the Filippov sense (Gouzé & Sari 2002). The most ‘problematic’ situation that arises in differential inclusions is a black wall—a boundary between a pair of orthants within each of which the solution to equation (2.1) is converging towards a point in the other orthant. Upon reaching the boundary from either direction, the solution cannot continue, as that would violate the dynamics of the other orthant. Instead, solutions in the Filippov sense remain on the wall, where they may nevertheless continue in some direction along the boundary, a phenomenon called a sliding mode. Since our formulation of the equations requires no self-input, and places restrictions on parameters, we are able to largely avoid these problems. As shown below, any two orthants for which all but one of the species are in the same qualitative state are separated by a transparent wall—not a black or even white wall. However, a further difficulty arises if two different species, *x*_*i*_ and *x*_*i*′_, reach their threshold concentrations, *θ*_*i*_ and *θ*_*i*′_, at exactly the same time. Then solutions may not be well-defined by virtue of continuation. That is, it is possible to have black walls that are of dimension *N* − 2 in 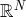. We will simply rule out this situation by restricting attention to trajectories in which no two concentrations ever reach their thresholds at the same instant. With that final assumption, solutions are well-defined and unique.

Because of the very special form of equation (2.1), and with the assumptions above, it is easy to calculate solutions from a given initial condition that is within one of the orthants. The solution is equal to that of a system of ordinary linear differential equations up until the time, if any, at which the first *x*_*i*_ reaches its threshold *θ*_*i*_. At this *switching* time, the solution switches to that of the linear equations given by the next orthant, and so on. The total trajectory can be pieced together as a sequence of solutions over constituent time intervals. Some features of the dynamics can be computed very efficiently. For example, it turns out that the map relating **x**(*t*) at a time when it crosses a particular boundary between orthants to the value **x**(*t*′) at the next time the trajectory crosses the boundary is a linear fractional map. Because the composition of two linear fractional maps is a linear fractional map, powerful results concerning the dynamic properties can be derived (Glass & Pasternack 1978*b*; Mestl *et al.* 1995; Edwards & Glass 2005).

## 3. The *N*-cube

A given trajectory of the system, **x**(*t*), passes through a discrete sequence of orthants with corresponding qualitative state vectors **X**^0^, **X**^1^, **X**^2^,…. We identify the 2^*N*^ possible qualitative state vectors **X** with the vertices of the *N*-cube. Because we have assumed that at each switching time precisely one *x*_*i*_ is crossing its threshold, *θ*_*i*_, then each successive qualitative state in the sequence **X**^0^, **X**^1^, **X**^2^,… differs from the previous state in exactly one position. The sequence can thus be viewed as a walk on the *N*-cube.

Further, the assumptions made above imply that each edge of the hypercube can be traversed in one and only one direction. To see this, let **X** and **X**′ be two qualitative state vectors that differ only in the *i*th position. Because *i*∉*R*_*i*_, we must have *X*_*i*_1__ = *X*′_*i*_1__, *X*_*i*_2__ = *X*′_*i*_2__,…,*X*_*i*_*k*_*i*___ = *X*′_*i*_*k*_*i*___. Thus, we must have *f*_*i*_(*X*_*i*_1__,*X*_*i*_2__,…,*X*_*i*_*k*_*i*___) = *f*_*i*_(*X*′_*i*_1__,*X*′_*i*_2__,…,*X*′_*i*_*k*_*i*___). Call this value *f*. Suppose that *λ*_*i*_*f*/*γ*_*i*_ > *θ*_*i*_. If *x*_*i*_ starts out below *θ*_*i*_, then it may cross *θ*_*i*_ as it increases towards *λ*_*i*_*f*/*γ*_*i*_. (It may not end up crossing, however, if some other *x*_*j*_ crosses its threshold *θ*_*j*_ first, changing the dynamics of *x*_*i*_.) If *x*_*i*_ starts out above *θ*_*i*_, then it converges monotonically towards *λ*_*i*_*f*/*γ*_*i*_, and so species *i* cannot change the qualitative state. A symmetric argument applies if *λ*_*i*_*f*/*γ*_*i*_ < *θ*_*i*_, so that, either way, the edge between the hypercube vertices corresponding to **X** and **X**′ can be traversed in one and only one direction. As we have assumed that *λ*_*i*_/*γ*_*i*_ > *θ*_*i*_, that direction is determined solely by the function *f*_*i*_.

Thus, the qualitative dynamics of a network with *N* genes can be represented by a directed graph on the *N*-cube. In this graph, there are *N* × 2^*N*−1^ edges. The truth table representing the control of production for each variable specifies a 1 or 0 for each of the 2^*N*−1^ states of the potential *N* − 1 inputs. In general, there is thus a 1:1 correspondence between directions of edges on the state transition diagram and entries in the truth tables, so that given one, the other can be generated. This observation forms the basis for solving the inverse problem.

Because each hypercube vertex is adjacent to *N* others, the total number of incoming and outgoing edges is *N* for every vertex. One important special case is a vertex with only incoming edges. In this case, the corresponding orthant contains a stable fixed point, and trajectories of the differential equations, if inside the orthant, can never leave it. Moreover, it should be clear that this behaviour is robust to small perturbations in the parameters *λ*_*i*_ and *γ*_*i*_, as well as to small perturbations to the system state **x**, as long as the state stays strictly within the orthant. For example, an abstract model of a genetic ‘toggle-switch’ (Glass & Kauffman 1973; Gardner *et al.* 2000), which includes two mutually repressive genes, would have two such orthants: one with gene 1 high and gene 2 low (10), and one with gene 1 low and gene 2 high (01). Each orthant has two incoming edges because if both start out low (00), then both concentrations would be growing, and one or the other would cross its repression threshold first, corresponding to the transition 00 → 10 or 00 → 01. Likewise, if both are high (11), then each would be repressing the other, but one would fall below its repression threshold first, corresponding to the transition 11 → 10 or 11 → 01.

**Figure 1.**
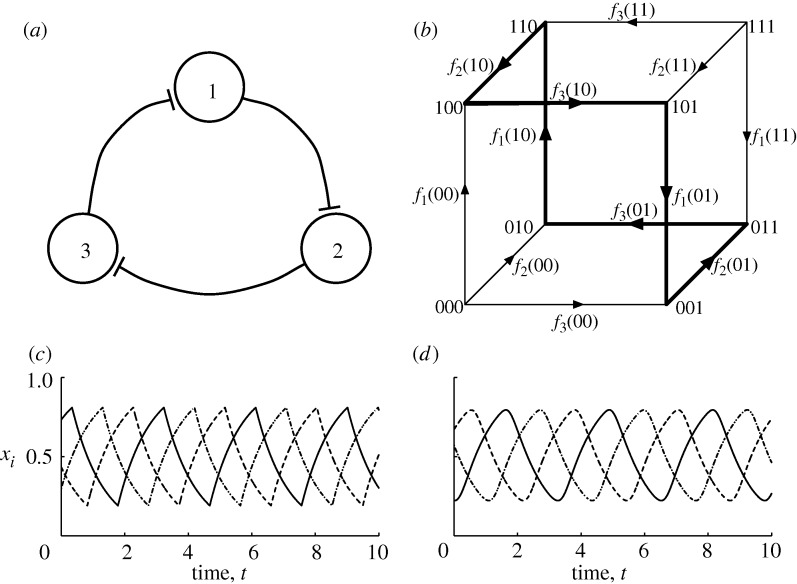
Analysis of the three-gene repressilator system described in table 1 and shown in panel (*a*). The state transition diagram in (*b*) has a one-to-one correspondence between edge directions and truth-table values as indicated by the edge labellings. The dynamics in equation (3.1) with (*c*) 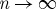 and (*d*) *n* = 10. *x*_1_, solid line; *x*_2_, dashed line; *x*_3_, dashed-dotted line.

We are particularly interested in cyclic behaviour, which brings up another important special case: a vertex with only a single outgoing edge. In such an orthant, the dynamics must eventually enter the orthant corresponding to the vertex to which the edge is directed. If there is a sequence of such vertices, each pointing to the next and with the last pointing back to the first, then the qualitative dynamics contain what we call a robust cycle. A trajectory beginning in any orthant along the cycle will stay on the cycle, repeatedly passing through each orthant. As with the stable orthants, this qualitative behaviour is robust to sufficiently small variations in the parameters *λ*_*i*_ and *γ*_*i*_, and to perturbations in the initial state or trajectory itself, as long as the perturbation does not change the orthant. A paradigmatic example of a robust cycle is seen in the repressilator network (figure 1*a*; Glass & Pasternack 1978*b*; Elowitz & Leibler 2000), a three-gene network in which each gene represses the next in line. The basic regulatory logic behind the repressilator is shown in table 1. The *N*-cube transitions are shown in figure 1*b*, where the robust cycle is highlighted in bold. In a typical, non-logical modelling treatment, simplified differential equations describing the dynamics of this network might be written using Hill functions as
3.1
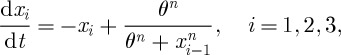

where *x*_0_ = *x*_3_, *θ* = 0.5 and *n* is positive. In the limit 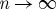, equation (3.1) is a piecewise linear equation of the form (2.1), and shows stable robust limit cycle oscillations with period 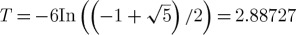
…. The dynamics are shown in figure 1*c*,*d*. Although in this case the robust cycle is globally attracting, as can be seen from figure 1*b*, this need not be so in general.

**Table 1.** Truth-table representations of the three regulatory functions for the repressilator, shown in figure 1. The one-to-one correspondence between truth-table components and edges in the three-cube is explicitly shown in figure 1*b*.

*X*_2_	*X*_3_	*f*_1_(*X*_2_*X*_3_)	*X*_1_	*X*_3_	*f*_2_(*X*_1_*X*_3_)	*X*_1_	*X*_2_	*f*_3_(*X*_1_*X*_2_)
0	0	1	0	0	1	0	0	1
0	1	0	0	1	1	0	1	0
1	0	1	1	0	0	1	0	1
1	1	0	1	1	0	1	1	0

## 4. Estimating regulatory architecture and functions

Historically, much of the focus of algorithms and analysis of the inverse problem has been on determining the regulators and regulatory functions, a problem which we will more concisely refer to as regulatory inference. This stands in opposition to inferring the real-valued parameters, *γ*_*i*_, *λ*_*i*_ and *θ*_*i*_. There are several reasons why this emphasis emerged as the focus of reverse engineering efforts. One is that these qualitative features of regulatory networks—which species regulate which and in what manner (e.g. promoting or blocking production)—are the primary interest of biologists and may be amenable to direct experimental observation. Morever, until recently, accurate quantitative data have rarely been available for regulatory network inference. There have been some exceptions to this rule, such as with the work of Reinitz and co-workers (Mjolsness *et al.* 1991; Reinitz & Sharp 1995; Jaeger *et al.* 2004), who began collecting and modelling fluorescence expression data on networks of developmental genes in *Drosophila melanogaster* in the early 1990s. When quantitative data have been available, it has often been a fairly direct measurement of some relevant biochemical parameter, such as the degradation rate of chemical species (our *γ*_*i*_), binding energies or binding frequencies (Bachmair *et al.* 1986; Belle *et al.* 2006). Thus, the possibility of direct measurement of real-valued model parameters seemed to obviate the need for incorporating their estimation into a more general network inference procedure. Finally, some modelling efforts have concluded that the dynamics of gene-regulatory networks may be robust to variations in parameters. For example, Von Dassow *et al.* (2000) showed that the qualitative behaviour of a model of the segment polarity network of *D. melanogaster* is highly robust to parameter variation. Subsequent studies have attributed this to specific feedback loops in the regulatory architecture (Albert & Othmer 2003; Ma *et al.* 2006). Placing this earlier work in the context of the current paper, we reiterate that specific state transition diagrams will lead to robust dynamical behaviour for a wide range of parameter values.

### (a) Edges and inference

Regulatory inference for models of the form of equation (2.1) has been studied under a number of different assumptions regarding the form of data available, including: noiseless, fully or partially observed, continuous-time trajectories (Glass & Young 1979; Perkins *et al.* 2004), periodic or ‘random’ observations of the *x*_*i*_ and their time derivatives (Glass *et al.* 2005; Perkins *et al.* 2006; Ichinose *et al.* 2008; Perkins & Hallett 2010; Porreca *et al.* 2010) or simply qualitative state vector sequences (Wilds & Glass 2009). Importantly, all of these results, many of which we will summarize shortly, can be understood as variations on how we obtain the one fundamental datum of regulatory inference: the direction of one edge on the *N*-dimensional hypercube describing the qualitative dynamics.

If we know the direction of every edge on the hypercube, then we can compute, that is, deduce, the corresponding *R*_*i*_ and *f*_*i*_. Recall that if *j* is a regulator of *i*, then there must be a setting for the qualitative states of all the other regulators of *i*, if any, such that changing the value of *X*_*j*_ changes the value of *f*_*i*_. By extension, there must be two qualitative states *X* and *X*′ that differ only in position *j* for which *f*_*i*_ has different values—that is, the edge describing *x*_*i*_’s qualitative dynamics is ingoing in one case, and outgoing in the other. Conversely, if no such pair *X* and *X*′ exists, then *j* is not a regulator of *i*. Thus, by checking all such pairs, one can determine definitively whether or not *j* regulates *i*. This procedure is computationally intensive, as it takes *O*(2^*N*^) time to check whether any particular *j* regulates any particular *i*, for a total computation time of *O*(2^*N*^*N*^2^). Having determined the regulators of each species, filling in truth tables describing the *f*_*i*_ takes time 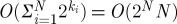. The procedure also requires a potentially large amount of data—one bit, or direction, for each edge of the *N*-dimensional hypercube, of which there are 2^*N*−1^*N*. Unless *N* is quite small, this approach thus requires a large amount of data and computation. In fairness, one cannot expect to do much better in general. For example, suppose that species *i* depends on all the other *N* − 1 species and *f*_*i*_ = 0 except for one particular combination of regulator states. Without an *O*(2^*N*^) check of the hypercube, one could not be sure whether *i* has any regulators at all.

### (b) Minimal networks

In a more realistic situation, we assume that we know the direction of only some of the edges on the hypercube. In this case, one cannot definitively deduce correct *R*_*i*_ and *f*_*i*_. A number of authors have suggested looking for candidate regulators *R*′_*i*_ and regulatory functions *f*′_*i*_ that are: (i) consistent with the given hypercube edge directions and (ii) a parsimonious explanation, in the sense of 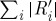 being minimal (Akutsu *et al.* 1999; Ideker *et al.* 2000; Perkins *et al.* 2006; Perkins & Hallett 2010).

Determining minimal-size *R*′_*i*_ that are consistent with a set of edge directions, *E* is a non-trivial computational problem. Importantly, each species can be treated independently, as every edge in the hypercube refers to a specific species changing qualitative state, and gives no information about other species. For species *i*, let *E*_*i*_ be the set of directed edges corresponding to a change in the qualitative state of species *i*. The most obvious way to find a minimal-size *R*′_*i*_ consistent with *E*_*i*_ is to start enumerating all possible *R*′_*i*_ in the order of increasing size. For any candidate, one can check if there is a function *f*′_*i*_, such that *R*′_*i*_ and *f*′_*i*_ together specify edge directions that agree with those found in *E*_*i*_. Akutsu *et al.* (1999) recommended doing this by enumerating all possible Boolean functions *f*′_*i*_ of the appropriate input dimensionality. However, as there are *O*(2^2^*k*^^) Boolean functions on *k* inputs, this is computationally awkward. A simpler approach, and equally correct, is to check if one can construct a consistent truth table *f*′_*i*_ using the candidate regulatory set *R*′_*i*_. Every edge (*X*,*X*′) in *E*_*i*_ implies that *f*′_*i*_ should be equal to *X*′_*i*_ for the qualitative regulatory state corresponding to *X* (or to *X*′, as the two will be the same). If two different edges imply different values for what *f*′_*i*_ should be in the same regulatory state, then no consistent *f*′_*i*_ is possible, and *R*′_*i*_ cannot be the correct regulatory set.

In terms of worst-case complexity, one cannot do much better than blind enumeration of increasing-size candidate regulator sets *R*′_*i*_. However, there is an approach that in practical computations can be vastly faster. For species *i*, suppose the edges *E*_*i*_ tell us that, in state *X*, *x*_*i*_ is converging to its low state, whereas, in state *X*′, *x*_*i*_ is converging to its high state. This difference in behaviour must be attributed to one or more regulators of *i* being in a different qualitative state. Thus, suppose we let *D*(*X*,*X*′) = {*j* ≠ *i*:*X*_*j*_ ≠ *X*′_*j*_} be the set of all species, not including *i*, that are in a different qualitative state when comparing *X* and *X*′. Then we must have that at least one element of *D*(*X*,*X*′) is a true regulator of *i*; that is, 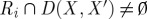. More generally, let 𝒳^0^_*i*_(*E*_*i*_) be the set of qualitative states (hypercube vertices) in which species *i* is converging towards its low state, according to the edges *E*_*i*_. Likewise, let 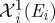 be the set of states in which species *i* is converging towards its high state. One can show that a candidate regulator set *R*′_*i*_ is consistent with the edges *E*_*i*_, if and only if 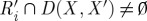, for every *X*∈𝒳^0^_*i*_(*E*_*i*_) and 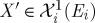. In general, finding a minimum-size set *S* that has non-empty intersection with a collection of other sets {*S*_1_,*S*_2_,…,*S*_*M*_} is called the set-cover problem (Ideker *et al.* 2000). The worst-case complexity for solving this problem is roughly *O*(*N*^*K*^), where the *S*_*i*_ are drawn from a set of size *N* and *K* is the size of the optimal solution. This can be considerably smaller than 2^*N*^ if *K* is sufficiently small. Further, when the *S*_*i*_ are small, they provide tight constraints on the solution *S*, and a minimum-size solution can often be found with considerably less computational effort.

### (c) The sample complexity of regulatory inference

If we can find minimal explanations consistent with a set of hypercube edge directions *E*, a natural question that arises is when does *E* contain enough information so that the minimal explanation is actually the correct one? In general, one cannot guarantee correctness unless all *N*2^*N*−1^ edge directions are known. Most analyses have made two additional assumptions: (i) that all species are regulated by precisely *K* others, where *K* may be much smaller than *N*, and (ii) that the data collection can be modelled by some simple random process, so that we can study expected data requirements rather than worst-case requirements.

The restriction on the size of regulatory sets, *K*, immediately gives us some traction. If we view each edge direction as providing one bit of information, then we can lower-bound the number of edge directions needed as 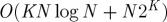 (Perkins *et al.* 2006). The first term describes the number of bits needed to specify the identities of the *K* regulators of all *N* species. The second term describes the number of bits needed to specify the truth tables of the *N* regulatory functions *f*_*i*_. The question, then, is whether any reasonable model of data collection can bring us close to this lower-bound.

Akutsu *et al.* (1999) studied Boolean network inference under the assumption of uniformly randomly sampled states. In the present context, this same result can be applied to a model in which we choose vertices of the hypercube uniformly randomly, and, for each vertex we choose, we are told the directions of all *N* adjacent edges. Akutsu and co-workers showed that the expected number of such samples that need to be taken before the minimal network is the correct one is 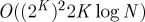, so that the expected number of edge directions is *O*((2^*K*^)^2^2*KN*log*N*). Perkins *et al.* (2006) provided evidence that this bound is loose, particularly regarding the (2^*K*^)^2^ term, and in a subsequent paper provided a tighter bound of 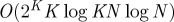 edge directions (Perkins & Hallett 2010)—more in line with the lower-bound.

At the opposite spectrum from uniformly random observations, Perkins *et al.* (2004) studied a situation in which data come from a sequence of vertices generated by the standard undirected walk on the *N*-cube. This was intended as a model of data generated from a real qualitative trajectory, although, as pointed out above, in the real qualitative dynamics each edge can be traversed in only one direction. Under this model, it was estimated that 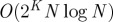 such qualitative states, or *O*(2^*K*^*N*^2^log*N*) observed edge directions, are needed before the minimal explanation is the correct one. The main difference compared with uniform random sampling is the 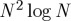-dependence of the number of edges needed, as opposed to *N*log*N*. In fact, there is a continuous spectrum between these two opposites. If data come from a series of qualitative states, where each is generated from the last by resetting the states of *d* of the *N* species, then 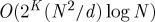 edges are needed in expectation. In summary, then, under these simple random-process models of data generation, the expected amount of data needed scales approximately as 2^*K*^, as suggested by the lower-bound, and as something between 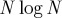 and *N*^2^log*N*, depending on how similar successive samples are in the Hamming distance sense.

## 5. Inferring regulation of the cell cycle in yeast

The cell cycle is the fundamental process by which cells multiply, in order that a multicellular organism or a colony of unicellular organisms may grow. There are many stages and processes involved in the cell cycle, including growth of the size of the cell, duplication and separation of the DNA, separation of the cytoplasm, and division of the cell into two daughter cells. We consider the cell cycle in yeast as analysed by Li *et al.* (2004). For more recent logical models and further references, see Fauré *et al.* (2009) or Fauré & Thieffry (2009). In yeast, the cell cycle is regulated by a core system of several genes. In carrying out the analysis, we take as variables Cln12 (representing cyclin transcription factors Cln1 and Cln2), Clb56 (representing Clb5 and Clb6), Clb12 (representing Clb1 and Clb2), Cdc20, Cdh1 and the size of the cell. We treat some pairs of factors as one because their empirical behaviour is highly correlated. As such, one could not meaningfully say that one or the other turns on first. Any regulatory effects we attribute to such a pair should be considered to represent an effect from one or the other gene, or possibly both.

Based on the earlier work by Li and Tang, as well as the work from his own laboratory, John Tyson has generated a qualitative sequence of high and low states for these variables, as shown in table 2. The data specify a single cycle of length 12 on the six-dimensional hypercube describing the qualitative dynamics of the cell cycle. Thus, the data give us the directions of just 12 edges out of the 192 total edges on the hypercube. For each of the six variables, only two of those edges tell us something definitive about the dynamics. For example, Cln12 increases from 0 to 1 on the transition from step 2 to step 3. Thus, we know that *f*_Cln12_ = 1 in step 2 (state 000011). Similarly, Cln12 decreases from 1 to 0 on the transition from step 6 to step 7, implying that *f*_Cln12_ = 0 in step 6 (state 111001). Cln12 does not change state between step 1 and step 2, for example, but this does not tell us the value of *f*_Cln12_ in step 1; the real, underlying concentration of Cln12 protein might be increasing or decreasing.

**Table 2.** Sequence of on/off (1/0) states for the six components of the yeast cell cycle model. The sequence repeats itself (1 comes after 12). This simplification has been provided by John Tyson, based on the work by Li *et al.* (2004).

step	Cln12	Clb56	Clb12	Cdc20	Cdh1	size
1	0	0	0	0	1	0
2	0	0	0	0	1	1
3	1	0	0	0	1	1
4	1	0	0	0	0	1
5	1	1	0	0	0	1
6	1	1	1	0	0	1
7	0	1	1	0	0	1
8	0	1	1	1	0	1
9	0	0	1	1	0	1
10	0	0	1	1	1	1
11	0	0	1	1	1	0
12	0	0	0	1	1	0

On the other hand, suppose we assume that the observed cycle is robust as described in §3, so that every vertex of the *N*-cube that is one step away from the cycle points to the cycle. Then, another 4 × 12 = 48 edges are implied by the data in table 2, because, for each state on the cycle, there are four adjacent states that are not part of the cycle. For example, state 100010 is not part of the cycle, but it is only one bit different from state 000010, which is the first step of the cycle. Thus, with the robustness criterion, we assume that the hypercube has a directed edge from state 100010 to state 000010.

We found minimal networks to explain both sets of data—the 12 edges implied by the cycle, and the 60 edges implied by the cycle and the robustness assumption. The results are shown in tables 3 and 4. Using only the cycle data, and not the robustness assumption, each variable can be explained by several other individual variables. For example, Cln12 turning on at step 3 and off at step 7 could be explained by the regulatory function *f*_Cln12_ = ¬Clb56 (i.e. Clb56 represses Cln12). In this explanation, Cln12 can turn on at step 3 because Clb56 is off and thus not repressing it, whereas Cln12 turns off at step 7 because Clb56 is on and actively repressing it. But then, the two switches in Cln12 can just as well be explained by *f*_Cln12_ = ¬Clb12 or by *f*_Cln12_ = Cdh1. Combining different minimal explanations for each variable, there are 3 × 3 × 5 × 4 × 3 × 2 = 1080 different minimal networks explaining the entire cycle—most of which are incorrect, in comparison with the known regulatory interactions between the variables.

**Table 3.** Alternative regulatory explanations of each variable using the set of edge directions specified by the cycle of states in table 2. All explanations are minimal in terms of the number of distinct regulating variables consistent with the edge set, and are given in terms of a logical formula for the regulatory function *f*. The symbol ¬ represents logical negation. Alternative minimal explanations are separated by a slash ‘/’.

gene	minimal regulation inferred from the cycle only
Cln12	¬Clb56/¬Clb12/Cdh1
Clb56	Clb12/¬Clb12/¬Cdc20
Clb12	Cln12/Clb56/¬Cdc20/¬Cdh1/size
Cdc20	Clb56/Clb12/¬Cdh1/size
Cdh1	¬Cln12/Clb12/Cdc20
size	¬Clb12/¬Cdc20

**Table 4.** Regulatory structure for the robust, minimal network inferred from the on/off state sequence in table 2 and the correct logic functions. The robustness and minimality requirements result in a network that is completely specified except for the precise form of the logic function for regulation of Clb12 (there are three alternative choices that are also consistent).

*i*	*f*_*i*_	‘correct’ *f*_*i*_
Cln12	¬Clb12 ∧ size	¬Clb12 ∧ size
Clb56	¬Cdc20 ∧¬Cdh1	¬Cdc20 ∧¬Cdh1
Clb12	Clb56 ∨ (Cdc20 ∧ size)	(¬Cdh1 ∨ size) ∧ (Clb56 ∨ Clb12)
Cdc20	¬Cln12 ∧ Clb12	¬Cln12 ∧ Clb12
Cdh1	¬Cln12 ∧¬Clb56	¬Cln12 ∧¬Clb56 ∧ (¬Clb12 ∨ Cdc20)
size	¬Cdc20 ∨¬Cdh1	¬Cdc20 ∨¬Cdh1

**Figure 2.**
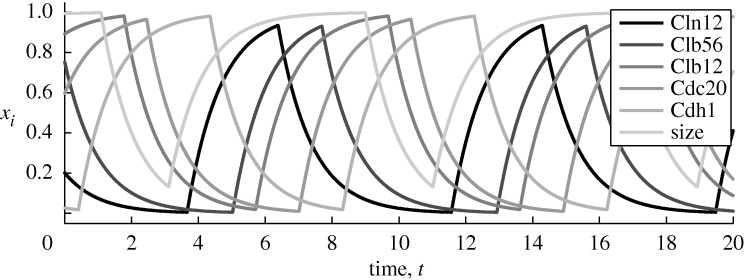
Integrated dynamics of the robust, minimal network identified from the on/off state sequence in table 2 with all thresholds (*θ*_*i*_) fixed at 0.5 and all degradation rates (*γ*_*i*_) set at 1. The explicit regulatory structure of the minimal network is shown in table 4.

In contrast, when we assume robustness, it turns out that there is a single minimal explanation for every variable, as shown in table 4. The formulae obtained also turn out to be much closer to the current biological understanding of yeast cell cycle regulation (John Tyson 2009, personal communication). The formulae for Cln12, Clb56, Cdc20 and size are exactly correct. For Cdh1, the correct explanation is only a minor refinement of the inferred rule. For Clb12, the inference procedure gets two of the four actual regulators correct (Clb56 and size), while missing Cdc20 and self-input from Clb12 (which was disallowed in the inference procedure). Thus, even with the robustness assumption, some incorrect inferences have been made. However, the results are far more accurate than without the robustness assumption. Figure 2 shows the dynamics produced by the inferred regulatory rules shown in table 4, assuming all *γ*_*i*_ = *λ*_*i*_ = 1 and *θ*_*i*_ = 0.5. The trajectory correctly reproduces the desired sequence of qualitative states.

The putative formulae on the right side of table 4 were obtained by human (i.e. non-automated) analysis of a large amount of extra data, including observations of what happens to the cell cycle when certain genes are artificially turned on or off. Such deletion and overexpression studies are the norm in genetics, precisely because it is often hard to infer causal links based on wild-type behaviour alone. Thus, we consider that the performance of the inference procedure, in conjunction with the robustness assumption, is very good on this example. It remains to be seen what would be inferred if deletion or overexpression-type data were added to that in table 2.

## 6. Conclusions

The main concept in the current paper is that the underlying logical structure of genetic control networks determines robust dynamics. The key device is to start with a symbolic sequence of Boolean state vectors in which each vector in the sequence differs from the previous in only one position. In our formulation, the sequence corresponds to a symbolic representation of concentrations, with 1 representing a high concentration and 0 representing a low concentration. If the Boolean vector sequence describes a cycle, with each unique vector occurring only once, then it is possible to generate a class of differential equations of the form of equation (2.1) that will guide the trajectory through the state space in the specified order (Glass & Pasternack 1978*b*; Ben-Hur & Siegelmann 2004). Although the desired dynamics are assured only if these differential equations incorporate Boolean functions, we have found that, by approximating the Boolean functions by appropriate continuous nonlinear sigmoidal functions, the essential features of the dynamics are generally preserved (Glass & Pasternack 1978*a*; Wilds & Glass 2009). Rigorous mathematical results are still needed to better define the actual limits of the robust dynamics.

An alternative, and perhaps more traditional, way to think about these networks starts not from the symbolic sequence of states through phase space, but rather from the structure of the underlying network—which elements activate or inhibit other elements in the network. For example, the well-known repressilator network, figure 1 (Elowitz & Leibler 2000), embodies a negative feedback circuit with three elements—an architecture that has long been recognized to generate stable robust oscillations (Kling & Székely 1968; Glass & Pasternack 1978*a*; Lewis & Glass 1992). The extent to which logical structures with robust symbolic sequences in the sense of the current paper may also underlie the robust dynamics observed in other mathematical models of genetic networks (Von Dassow *et al.* 2000; Albert & Othmer 2003; Ma *et al.* 2006) remains to be determined.

As knowledge accumulates about biological structure, dynamics and interactions, it easy to be overwhelmed by the incredible complexity that appears evident at all levels of organization. A fundamental question is whether there will be simplifying principles that will help us to develop conceptual and computational insights. We believe that the current formulation, which relates the underlying logical structure of biological networks to robust dynamics, provides one potentially important approach. Although the observation that we were able to reproduce many of the currently accepted interactions for the yeast cell cycle based on the concepts of robustness and minimality offers support for our approach, we believe that we are still at the very beginning of exploring these ideas.
